# Permanent endoscopic transpapillary gallbladder drainage using a novel spiral stent (IYO‐stent)

**DOI:** 10.1002/deo2.40

**Published:** 2021-09-02

**Authors:** Yuichi Takano, Jun Noda, Masataka Yamawaki, Tetsushi Azami, Takahiro Kobayashi, Fumitaka Niiya, Naotaka Maruoka, Tatsuya Yamagami, Masatsugu Nagahama

**Affiliations:** ^1^ Division of Gastroenterology Department of Internal Medicine Showa University Fujigaoka Hospital Kanagawa Japan

**Keywords:** acute cholecystitis, endoscopic transpapillary gallbladder drainage

## Abstract

**Background and aim:**

Endoscopic transpapillary gallbladder drainage (ETGBD) is widely performed. However, there is no consensus on the appropriate diameter, length, and shape of the stent that should be used in this procedure. In addition, there are limited data on the outcomes of permanent ETGBD. In our facility, a stent with a novel spiral structure (IYO‐stent) is permanently placed in patients with acute cholecystitis who are not indicated for surgery. This study examined the efficacy and safety of the IYO‐stent in cases of permanent ETGBD.

**Methods:**

We retrospectively examined patients who underwent permanent ETGBD using the IYO‐stent from April 2018 to December 2020.

**Results:**

Eleven patients were included in this study. The technical success and the clinical success rate were 91%. One patient had a post‐procedure adverse event (post‐endoscopic sphincterotomy bleeding). Within the median observation period of 312 days (range: 109–742), late adverse events, including cholangitis (*n* = 1) and incomplete stent migration (*n* = 1), were observed. However, none of the patients experienced cholecystitis relapse.

**Conclusion:**

Permanent ETGBD with IYO‐stent is an effective treatment for the patients with acute cholecystitis who are not indicated for surgery.

## INTRODUCTION

Cholecystectomy is the curative treatment for acute cholecystitis.^1^, ^2^ However, gallbladder drainage is performed on patients at high risk for surgery or from whom consent cannot be obtained.^3^ The different types of gallbladder drainage are percutaneous transhepatic gallbladder drainage (PTGBD), endoscopic transpapillary gallbladder drainage (ETGBD), and endoscopic ultrasound‐GBD.^3^, ^4^

ETGBD is a procedure that applies endoscopic retrograde cholangiopancreatography (ERCP) technique. Conventionally, a biliary plastic stent is placed in the gallbladder. In recent years, stents dedicated to gallbladder drainage have been developed.^5^, ^6^, ^7^ However, there is no consensus on the appropriate diameter, length, and shape of stents that should be used in ETGBD. In addition, there are limited data on the outcomes of permanent ETGBD.

**FIGURE 1:**
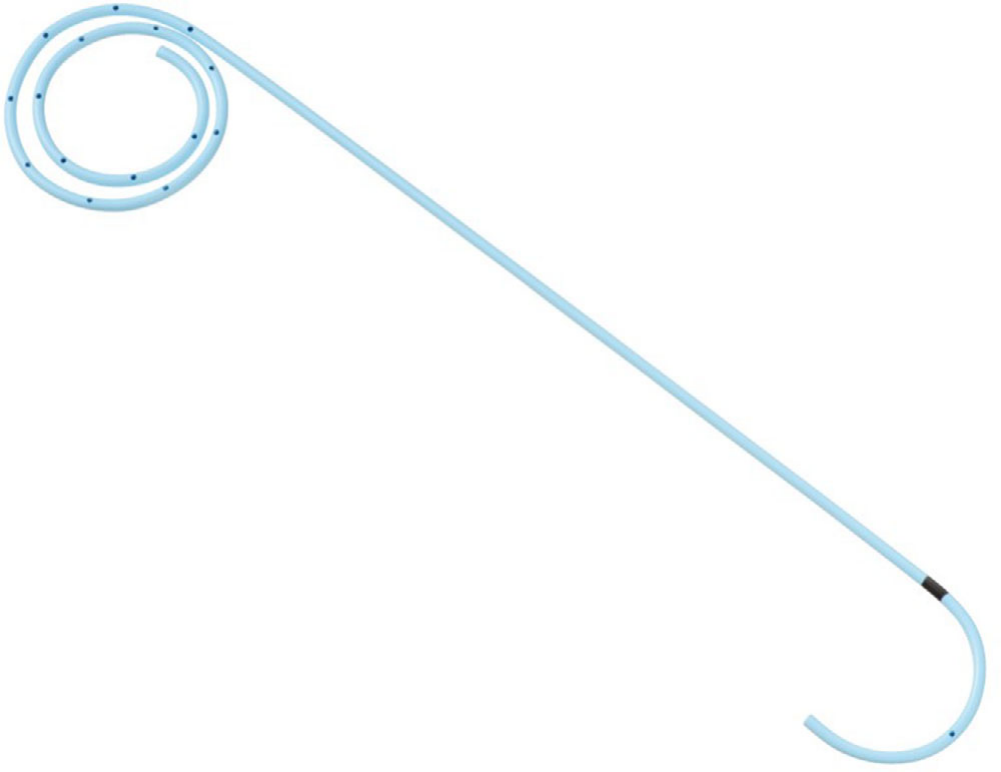
A 5‐Fr transpapillary gallbladder stent (IYO‐stent). The stent has only one size (diameter: 5 Fr, length: 32 cm) and has a novel spiral structure. The spiral part has a circular diameter of 3 cm and has 36 side holes. There are six side holes on the duodenal side

Since 2018, our facility has been performing permanent ETGBD using the unique spiral stent (IYO‐stent, Gadelius Mediclal, Tokyo, Japan) in patients with acute cholecystitis who are not indicated for surgery. The stent has only one size (diameter: 5 Fr, length: 32 cm) and has a novel spiral structure (Figure 1).

## AIM

This study examined the efficacy and safety of the IYO‐stent in permanent ETGBD.

## METHODS

### Study design

Patients who underwent ETGBD using the IYO‐stent from April 2018 to December 2020 were retrospectively examined using information collected from the medical records. Data, including clinical backgrounds, technical and clinical success rates, adverse events, and long‐term prognosis, were examined. The study (case series) was performed with the approval from the Showa University ethics committee (Approval number: F2021C009).

### Indications for ETGBD

The patients with grade II (moderate) or grade III (severe) acute cholecystitis according to the Tokyo guidelines 2018 (TG18) and who had no indication for surgery were enrolled. Among the cases enrolled, PTGBD is difficult (massive ascites, bleeding tendency, removal risk due to dementia, Chiladiti syndrome), or internal drainage from PTGBD is required (cholecystitis relapses after PTGBD clamping, history of multiple cholecystitis) was indicated for ETGBD.

The cases in which ETGBD was directly performed for acute cholecystitis were defined as one‐step group, and the cases in which ETGBD was performed after PTGBD or endoscopic naso‐gallbladder drainage (ENGBD) were defined as two‐step group.

### ETGBD procedure

**FIGURE 2:**
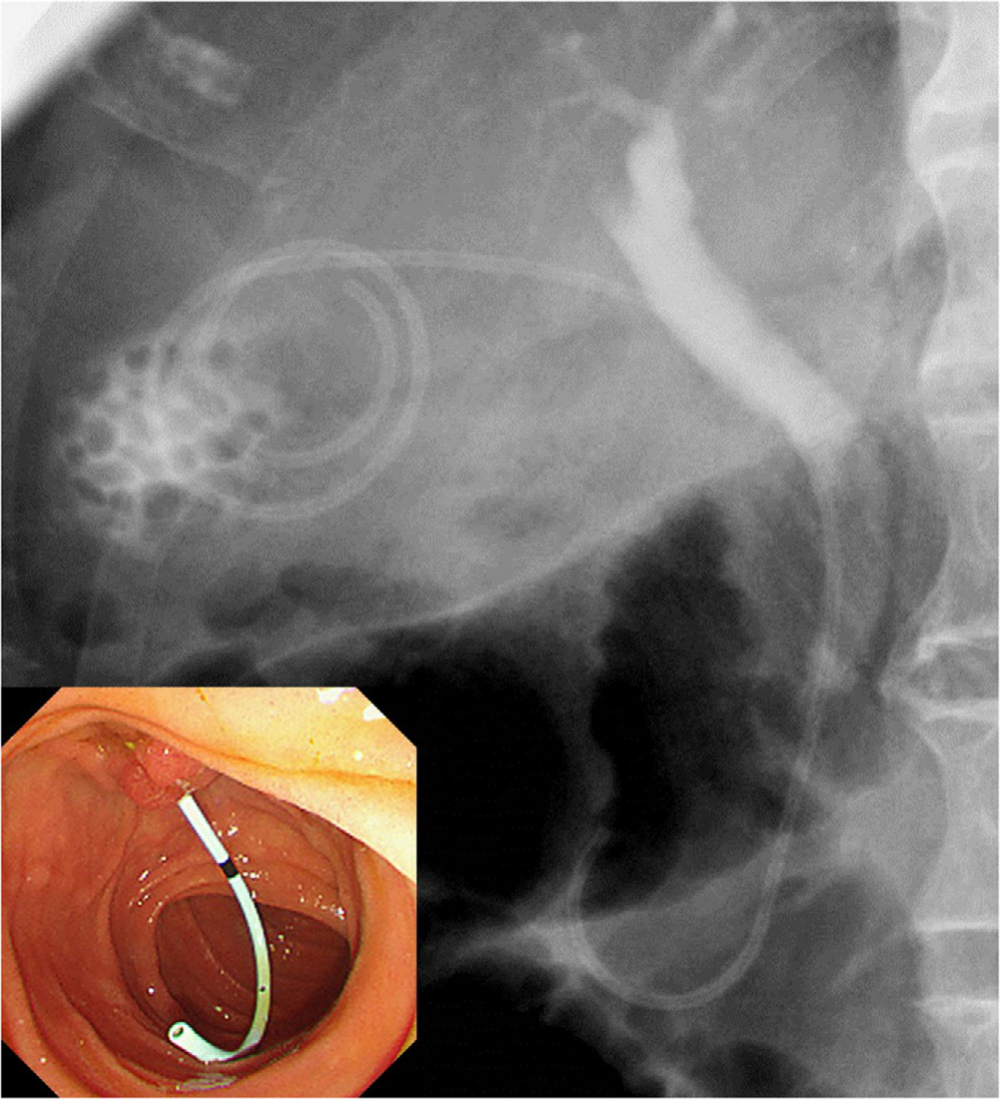
Endoscopic transpapillary gallbladder drainage using the IYO‐stent. Note the unique spiral shape in the gallbladder

An endoscope (JF‐260V, Olympus Medical Systems, Tokyo, Japan) was inserted into the duodenum, and a catheter (PR‐V614M, Olympus) was intubated from the papilla of Vater to the biliary duct. Endoscopic sphincterotomy (EST) or endoscopic papillary balloon dilation was performed if needed. In cases with common bile duct stones, endoscopic stone extraction was performed. The location of the cystic duct was confirmed via cholangiography, and the cystic duct was breached with a 0.025‐inch guidewire (Visiglide, Olympus), which was placed to form a loop in the gallbladder. Dilation was performed using the 6‐Fr Soehendra dilation catheter as needed. Finally, the IYO‐stent was placed in the gallbladder (Figure 2).

### Outcome measurements

Technical success was defined as placement of the IYO‐stent in the gallbladder. The clinical success of one‐step group, PTGBD and ENGBD was defined as improvement in clinical symptoms (abdominal pain/fever) of cholecystitis within 3 days after the procedure. Further, in the two‐step group, successful removal of the external drainage (PTGBD or ENGBD) without relapse of cholecystitis after IYO‐stent placement was defined as clinical success.

At our facility, cases that are not indicated for surgery are targeted for ETGBD. As stated in the clinical background of the patients, every case has an underlying disease. For this reason, ETGBD has a policy of permanent indwelling, and we will replace it on demand when cholecystitis or cholangitis develops.

Adverse events were defined as per the definition by the American Society of Gastrointestinal Endoscopy.^8^

## RESULTS

### Clinical background of patients

Eleven patients (nine men and two women) were included in this study. The median age was 79 (range: 63−85) years. All presented with gallbladder stones. Based on the severity grade of TG18, 10 patients were grade II (moderate), and one was grade III (severe). Five (45%) patients had common bile duct stones. The underlying diseases were unresectable malignant tumor (*n* = 3), dementia (*n* = 3), liver cirrhosis (*n* = 2), sequelae of cerebral hemorrhage (*n* = 1), chronic atrial fibrillation (*n* = 1), old myocardial infarction (*n* = 1), and Chiladiti syndrome (*n* = 1) (with duplication). Two patients presented with a surgically altered anatomy. One patient had undergone Billroth‐I reconstruction, and another underwent Billroth‐II reconstruction. There were five cases (45%) in the one‐step group, and six cases (55%) in the two‐step group. ENGBD was performed on one patient prior to the procedure, and five patients had indwelling PTGBD. Clinical success rate was 100% (6/6) in the ENGBD and PTGBD, and no adverse event was observed. Three patients (27%) received oral antithrombotic drug (Table 1).

**TABLE 1:** Clinical background of the patients

Number of patients	11
Age, median (range), years	79 (63−85)
Sex, male: female, no.	9:2
Gallbladder stone, no. (%)	11(100)
Common bile duct stone, no. (%)	4(36)
Severity grade of Tokyo guidelines 2018, no. (%)	
GradeⅡ	10(91)
GradeII	1(9)
Underlying diseases, no. (%)	
Unresectable malignant tumor	3(27)
Dementia	3(27)
Liver cirrhosis	2(18)
Sequelae of cerebral hemorrhage	1(9)
Chronic atrial fibrillation	1(9)
Old myocardial infarction	1(9)
Chiladiti syndrome	1(9)
Surgically altered anatomy, no. (%)	
Billroth‐I reconstruction	1(9)
Billroth‐Ⅱ reconstruction	1(9)
One‐step group, no. (%)	5(45)
Two‐step group, no. (%)	6(55)
ENGBD* prior to the procedure, no. (%)	1(9)
PTGBD^†^ prior to the procedure, no. (%)	5(45)
Clinical success of ENGBD and PTGBD, no. (%)	6(100)
Adverse events of ENGBD and PTGBD, no. (%)	0(0)
Oral antithrombotic drug, no. (%)	3(27)

* ENGBD: endoscopic naso‐gallbladder drainage.

^†^ PTGBD: percutaneous transhepatic gallbladder drainage.

### Clinical outcomes of the procedure

A guidewire manipulation to gallbladder was successful in all patients. The technical success rate of the procedure was 91% (10/11). In the unsuccessful case, the IYO‐stent could not advance into the gallbladder due to stones filling the gallbladder. The purpose of this case was internal drainage from PTGBD. We decided to place PTGBD permanently. The clinical success rate was 100% (5/5) in the one‐step group and 83% (5/6) in the two‐step group. The median procedure time was 41 min (range: 16–61). Common bile duct stones were all removed endoscopically (36%). The dilation with the 6‐Fr Soehendra dilation catheter was performed in three cases (27%). Post‐procedure (<14 days) adverse events rate was 9% (1/11). Post‐EST bleeding was observed 4 days after the procedure in one patient, and endoscopic hemostasis (clipping) was required. Other adverse events, including acute pancreatitis and cystic duct perforation by the guidewire, were not observed.

The median observation period in 10 patients who underwent successful stent placement was 312 (range: 109−742) days, and none of the patients experienced cholecystitis relapse. By contrast, one patient presented with acute cholangitis 201 days after placement, and one experienced incomplete stent migration 113 days after placement. Late (>14 days) adverse events rate was 20% (2/10) (Table 2).

**TABLE 2:** Clinical outcomes of the procedure

Technical success, no. (%)	10(91)
Clinical success	
One‐step group, no. (%)	5(100)
Two‐step group, no. (%)	5(83)
Procedure time, median (range), min	41(16‐61)
Stone extraction in the same session, no. (%)	4(36)
Previous endoscopic sphincterotomy(EST), no. (%)	3(27)
Endoscopic sphincterotomy, no. (%)	4(36)
Endoscopic papillary balloon dilation, no. (%)	4(36)
Dilation using the 6‐Fr Soehendra dilation catheter, no. (%)	3(27)
Postprocedure (<14 days) adverse events, no. (%)	
Post‐EST bleeding	1(9)
Pancreatitis	0(0)
Cystic duct perforation by the guidewire	0(0)
Stent migration	0(0)
Observation period, median days (range)	312 (109−742)
Late (>14 days) adverse events, no. (%)	
Cholecystitis	0(0)
Cholangitis	1(9)
Stent migration	1(9)

**FIGURE 3:**
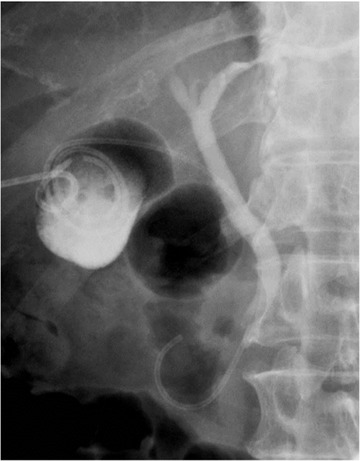
Immediately after placement, the lower end of the IYO‐stent was found in the second portion of the duodenum. Percutaneous transhepatic gallbladder drainage is already indwelled

**FIGURE 4:**
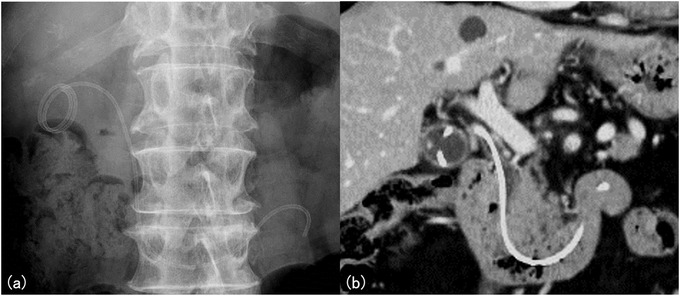
(a) A same case as observed in Figure 3. Plain radiography of the abdomen 3 months after the procedure. The distal end of the stent migrated to the third portion of the duodenum. (b) Computed tomography images revealed that the tip of the stent remained in the gallbladder. There was no cholecystitis relapse. We recommended stent replacement. However, the patient refused. Hence, follow‐up examination was performed

In case of acute cholangitis, common bile duct stone and debris were found by cholangiography. After removal of the IYO‐stent, stone and debris were removed with a balloon catheter. IYO‐stent was placed again, and cholangitis improved. In case of stent migration, plain abdominal radiography was performed at an outpatient department 3 months after placement, and the anal side of the stent had migrated to the third portion of the duodenum. Computed tomography (CT) confirmed that the tip of the stent remained in the gallbladder. The patient was asymptomatic and had no cholecystitis relapse. Since he refused to undergo stent replacement, follow‐up was continued (Figures 3 and 4).

## DISCUSSION

ETGBD is an established procedure and is listed in the TG18. It is classified into ENGBD, which is an external drainage, and endoscopic gallbladder stenting (EGBS), which is an internal drainage.^3^ There is no difference in terms of technical and clinical success and adverse events between ENGBD and EGBS.^3^, ^9^ The good indications for ETGBD include bleeding tendency, use of oral antithrombotic drugs, massive ascites, and Chilaiditi syndrome, which is not indicated for PTGBD.^10^ However, ETGBD is a challenging procedure, and easy enforcement should be avoided.^3^ Guidewire manipulation from the cystic duct to the gallbladder should be performed by a skilled endoscopist. Moreover, attention should be provided to adverse events, such as cystic duct perforation and acute pancreatitis.

The TG18 states that ETGBD may be considered in institutions with pancreaticobiliary endoscopy experts, but PTGBD should be the standard drainage in institutions where such experts are not available.^3^ In a recent meta‐analysis, the technical success of ETGBD was 83%, clinical success was 88.1%, and frequency of adverse events was 9.6%.^11^

Most of the difficulty in ETGBD is due to the complexity in using a guidewire in order to break through the cystic duct. Especially, in cases where the cystic duct draws an anatomically complex spiral, or the cystic duct is obstructed with impacted stones. Technically the tip here is to rotate the guidewire rather than pushing and pulling, and carefully breaking through the cystic duct. Replacement with a hydrophilic 0.035‐inch guide wire (Radifocus, Terumo, Tokyo, Japan) may be effective. If a conventional catheter does not point in the direction of the cystic duct, a catheter that can change directions (Swing Tip, Olympus Medical Systems, Tokyo, Japan) may also be effective.

Thus far, the biliary plastic stent was used in EGBS (mostly 7‐Fr, double pigtail type). However, in cases where the cystic duct is long, and the size of the gallbladder is large, the length of the biliary stent (usually up to 15 cm) is insufficient, and the stent cannot be placed in the fundus of the gallbladder. Inadequate length can cause stent migration and increase the risk of cholecystitis recurrence. In addition, the cystic duct usually draws a gentle curve, so a straight shaft of biliary stent may not fit its original shape.

Therefore, a stent dedicated to the gallbladder drainage has been developed. Inoue et al developed a gallbladder stent with a pigtail tip and a straight shape at the duodenal side.^5^ The authors hypothesized that the straight shape on the duodenal side would be effective in preventing stent dysfunction due to food residue.

Nakahara et al developed a newly designed stent for EGBS (GBest‐N stent; Hanaco Medical Co., Saitama, Japan).^6^ The length of the stent varies (11, 13, 15, 17, and 19 cm). The tip of the stent has a three‐dimensional spiral‐shaped structure, and there are side holes inside the spiral. The spiral‐shaped tip can prevent stent migration. The diameter of the stent shaft is 7 Fr, and its shape is semicircular to fit the nature curve of the cystic duct. They compared the outcomes between the GBest‐N stent (*n* = 23) and the conventional biliary stent (*n* = 47).^6^ The technical success rates were 100% and 95.7%. Hence, the result did not significantly differ. However, the incidence of late adverse events was significantly lower in the GBest‐N stent group (4.3%) than in the conventional biliary stent group (40.4%). In particular, the stent migration rates were 32% (15/47) in the conventional biliary stent group and 0% (0/23) in the GBest‐N stent group. The newly developed stent was effective in preventing stent migration.

The problem with EGBS is that the length of the cystic duct and size of the gallbladder significantly vary. The appropriate stent varies per individual.

Doi et al proposed a unique tailor‐made gallbladder stenting method using endoscopic naso‐biliary drainage (ENBD).^7^ They placed a 5‐Fr ENBD to the fundus of the gallbladder and measured the length between the Vater papilla and fundus of the gallbladder. The ENBD was cut to the measured length and was placed as an internal gallbladder drainage stent. EGBS was performed in 40 patients with acute cholecystitis, and the technical success rate of the procedure (stent placement success) was 75% (30/40). Cholecystitis improved in 29 of 30 indwelling EGBS cases, and the post‐procedure adverse events were cystic duct perforation (*n* = 1) and pancreatitis (*n* = 1). In total, 37 (92.5%) of 40 patients underwent elective cholecystectomy with a median waiting time of 42 (range: 12−138) days before surgery. No late adverse events, including stent migration, pancreatitis, and relapse of cholecystitis were observed. The advantage of this method is that a tailor‐made stent with appropriate length can be placed.

Interestingly, the median length between the Vater papilla and the fundus of the gallbladder was 25 (range: 17−35) cm.^7^ This is obviously longer than the stent usually placed in the gallbladder that is, a stent longer than the conventional biliary stent must be placed in the gallbladder. Appropriate placement in the gallbladder may help prevent adverse events, particularly stent migration.

In acute cholecystitis, the gallbladder swells due to inflammation. When ETGBD is successful, the gallbladder shrinks, and the stent is pushed out to the duodenal side. The gallbladder stent can migrate easily when it is placed in a shallow position. On the other hand, migration can be prevented when the stent is placed deeply in the gallbladder as the tip of the stent will remain in the gallbladder. Therefore, shallow placement of EGBS should be avoided.

In this study, one patient experienced stent migration. This case did not show complete migration, and CT scan revealed that the tip of the stent remained in the gallbladder. No cholecystitis relapse was observed. The unique feature of the IYO‐stent includes its spiral structure and the long length (32 cm). Due to the characteristics of IYO‐stent, complete stent migration may not likely occur. In addition, the stent has one size (diameter: 5 Fr, length: 32 cm). Thus, there is no need to select the stent length for individual cases.

Since the IYO‐stent has a small diameter of 5 Fr, it has an advantage that the stent can be inserted smoothly even in cases with impacted stones in the cystic duct or when the cystic duct is flexed strongly. Furthermore, the risk of pancreatitis is low because pancreatic duct expression at the papilla of Vater is unlikely to occur.

The technical and clinical success rates in this study were satisfactory at 91%. Relatively long‐term observation (median: 312 days) was possible, but no relapse of cholecystitis was found. Permanent ETGBD with IYO‐stent is an effective treatment for patients with acute cholecystitis who are not indicated for surgery.

Even after ETGBD, there is concern about stent occlusion due to debris, gallstones, and viscous bile juice. However, IYO stents are less likely to cause stent occlusion due to the large number of side holes on the gallbladder side. Another possibility is that it is preventing cystic duct obstruction. Recurrence of cholecystitis can be prevented if stone impaction on the cystic duct is avoided. IYO‐stent can be firmly placed in the cystic duct, and it may contribute to the prevention of relapse of cholecystitis.

The stent has some disadvantages. The stent and pusher are not integrated. Once the stent is inserted into the scope, it cannot be pulled back. In this study, there was one case in which the stent could not be advanced into the gallbladder, although the guidewire placement was successful. The integrated type stent can be pulled back, and biliary dilation with a dilator is possible; however, IYO‐stent cannot be pulled back. In cases where the gallbladder is filled with stones, sufficient dilation should be performed prior to gallbladder stenting. There seems to be no other case in which the IYO stent should be avoided.

There is no consensus in terms of the appropriate size, shape, length of EGBS, and studies are still ongoing. The IYO‐stent has a unique shape, which is not found in conventional gallbladder stents, and its long length is sufficient to be placed deeply in the gallbladder. Although the number of cases in this study was small, the IYO‐stent can be an option for permanent gallbladder drainage.

The current study had several limitations. The number of patients is limited. Moreover, the study was retrospective and conducted at a single center. In the future, larger studies should be conducted in multiple centers.

## CONFLICT OF INTEREST

The authors declare that they have no conflict of interest.

## FUNDING INFORMATION

None.
